# Calmodulin antagonists promote TRA-8 therapy of resistant pancreatic cancer

**DOI:** 10.18632/oncotarget.4490

**Published:** 2015-07-11

**Authors:** Kaiyu Yuan, Sun Yong, Fei Xu, Tong Zhou, Jay M McDonald, Yabing Chen

**Affiliations:** ^1^ Department of Pathology, University of Alabama at Birmingham, Alabama 35294, Birmingham, USA; ^2^ Department of Medicine, University of Alabama at Birmingham, Alabama 35294, Birmingham, USA; ^3^ Birmingham Veterans Affairs Medical Center, Alabama 35294, Birmingham, USA

**Keywords:** death receptor 5, apoptosis, resistance, calmodulin antagonists, pancreatic cancer

## Abstract

Pancreatic cancer is highly malignant with limited therapy and a poor prognosis. TRAIL-activating therapy has been promising, however, clinical trials have shown resistance and limited responses of pancreatic cancers. We investigated the effects of calmodulin(CaM) antagonists, trifluoperazine(TFP) and tamoxifen(TMX), on TRA-8-induced apoptosis and tumorigenesis of TRA-8-resistant pancreatic cancer cells, and underlying mechanisms. TFP or TMX alone did not induce apoptosis of resistant PANC-1 cells, while they dose-dependently enhanced TRA-8-induced apoptosis. TMX treatment enhanced efficacy of TRA-8 therapy on tumorigenesis *in vivo*. Analysis of TRA-8-induced death-inducing-signaling-complex (DISC) identified recruitment of survival signals, CaM/Src, into DR5-associated DISC, which was inhibited by TMX/TFP. In contrast, TMX/TFP increased TRA-8-induced DISC recruitment/activation of caspase-8. Consistently, caspase-8 inhibition blocked the effects of TFP/TMX on TRA-8-induced apoptosis. Moreover, TFP/TMX induced DR5 expression. With a series of deletion/point mutants, we identified CaM antagonist-responsive region in the putative Sp1-binding domain between −295 to −300 base pairs of DR5 gene. Altogether, we have demonstrated that CaM antagonists enhance TRA-8-induced apoptosis of TRA-8-resistant pancreatic cancer cells by increasing DR5 expression and enhancing recruitment of apoptotic signal while decreasing survival signals in DR5-associated DISC. Our studies support the use of these readily available CaM antagonists combined with TRAIL-activating agents for pancreatic cancer therapy.

## INTRODUCTION

Pancreatic cancer is the fourth leading cause of cancer death in the USA [1]. The 5-year survival rate has only improved from 2% to 6% in the past 30 years [2]. The only potentially curative therapy for pancreatic cancer is surgical resection. Unfortunately, even among those patients who undergo resection for pancreatic cancer and have tumor-free margins, the 5-year survival rate after resection is 10% to 25% [3]. Current chemotherapy, such as gemcitabine or 5-fluorouracil, coupled with radiotherapy may improve the quality of life of some patients, but their survival benefit is very limited [4]. Multi-drug resistance to chemotherapy is a major obstacle to obtaining a better prognosis for patients with pancreatic cancer.

Dysregulation of apoptosis of tumor cells plays an important role in the pathogenesis of pancreatic cancer and their resistance to therapies [5]. Apoptosis can be initiated either from intracellular signals via mitochondria through the intrinsic pathway, or from extracellular signals via plasma membrane receptors through the receptor-mediated extrinsic pathway [6]. The intrinsic pathway is mostly engaged by conventional chemotherapeutic drugs and radiation therapy [7]. On the other hand, the extrinsic pathway is initiated by activation of death receptors (DRs) present on the cell surface, including the tumor necrosis factor receptor (TNF-R), Fas death receptor (CD95), and TNF-related apoptosis inducing ligand (TRAIL) receptors [8]. The possibility of targeting TNF-R and Fas for tumor-specific killing has been limited due to systemic toxicity and lack of selectivity for tumors over normal tissues [9]. In contrast, TRAIL-induced apoptosis has been demonstrated in a wide variety of tumor cells *in vitro* and *in vivo*, and has been consistently highly selective for tumor cells over normal cells [10]. Many recombinant TRAIL or monoclonal antibodies to its receptors have been tested in phase I–III clinical trials for their anti-tumor efficacy. Among the antibodies for DR4 or DR5, conatumumab (AMG655, antibody for DR5) [11] and tigatuzumab (CS-1008/TRA-8, antibody for DR5) [12] are being tested for treatment of pancreatic cancers (http://Clinicaltrials.gov). In general, these agents have been well-tolerated, showing low toxicity in patients in several clinical trials [10, 13]. However, clinical trials with the TRAIL and DR4/5 agonist antibodies to date have shown limited anti-tumor efficacy. Preclinical studies have shown that many cancer cells are resistant to TRAIL-induced cell death, especially some highly malignant tumors such as pancreatic cancer [14]. Accordingly, resistance to TRAIL-induced apoptosis in cancer cells remains a serious clinical challenge. Better understanding of the molecular and cellular mechanisms of TRAIL resistance is critical for the successful application of TRAIL and DR4 or DR5 agonist antibodies in cancer therapy.

TRAIL-induced apoptosis is initiated by binding of TRAIL to its functional receptors (DR4 or DR5) that triggers the assembly of the death-inducing signaling complex (DISC), which in turn recruits the Fas-associated death domain (FADD) and eventually leads to recruitment and activation of initiating caspases at the DISC, including caspase-8 and -10 [15]. FADD may also recruit survival signals into the DISC, such as the enzymatically inactive homologue of caspase-8, FLICE-like inhibitor protein (FLIP), or Src kinase, and thus convert death receptor-activated apoptotic signals into survival signals [16]. In addition, modulation of other components in the death receptor-mediated signaling pathways, including increased expression of TRAIL decoy death receptors (DcR1 and DcR2), low expression or mutations of the functional receptors, DR4 or DR5, or over-expression of anti-apoptotic proteins, have been shown to contribute to the resistance of cancer cells to TRAIL therapy [10]. However, as many cancer cells express intact death receptors and the components of apoptotic signaling pathway [14, 17, 18], the mechanisms underlying the resistance of pancreatic cancer to TRAIL-induced apoptosis are not fully understood.

We have previously demonstrated that calmodulin (CaM) is recruited into the Fas death receptor-activated DISC and binds to the survival signals FLIP and Src in the DISC, thus mediating death receptor-mediated survival pathways [19–23]. CaM is a small calcium binding protein that interacts with a diverse group of cellular proteins and participates in signaling pathways that regulate proliferation, motility and differentiation [24]. We and others have shown that CaM antagonists induce apoptosis of cancer cells, including cholangiocarcinoma [21], lung adenocarcinoma [25] and breast carcinomas [26], via decreasing activation of AKT and increasing activation of caspase-8 or down-regulation of the anti-apoptotic Bcl-2 protein and increase of the pro-apoptotic Bax protein. In pancreatic cancer cells, we have demonstrated that CaM binding to Src in the DISC mediates the survival signals activated by the Fas death receptor signals [19]. The function of CaM in TRAIL-induced apoptosis is unknown. In the present studies, we characterize the role of CaM in TRA-8-induced apoptosis of resistant pancreatic cells and the underlying mechanisms. We demonstrated that CaM was recruited into DR5-activated DISC, which was inhibited by CaM antagonists, trifluoparazine (TFP) and tamoxifen (TMX). Although TFP or TMX alone did not induce apoptosis of pancreatic cancer cells, they markedly enhanced TRA-8-induced apoptosis of resistant pancreatic cells. Mechanistic characterization demonstrated that TFP or TMX inhibited the recruitment of the survival signal, Src, and increased recruitment and activation of caspase-8 in the DR5-activated DISC. Furthermore, CaM antagonists were found to increase the expression of DR5, which may further lead to enhanced DISC recruitment and activation of caspase-8. These studies reveal a novel function and underlying mechanisms of CaM in regulating TRA-8-induced apoptosis, and support the use of the readily available CaM antagonists to enhance therapeutic efficacy of TRAIL-resistant pancreatic cancer to TRAIL-agonist therapy.

## RESULTS

### CaM antagonists promote TRA-8-induced apoptosis in resistant pancreatic cancer cells

**Figure 1:**
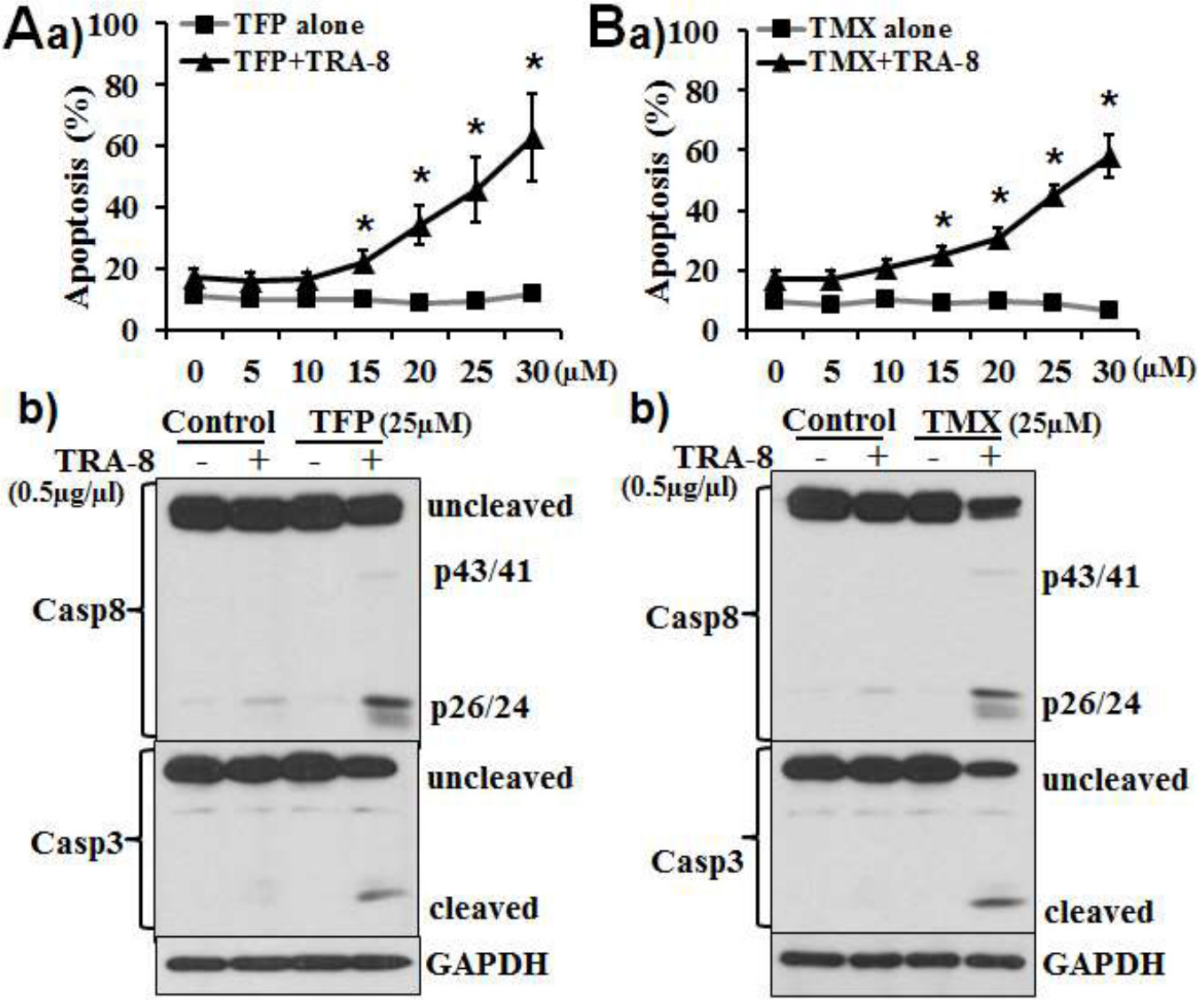
CaM antagonists promote TRA-8-induced apoptosis in TRA-8 resistant pancreatic cancer cells PANC-1 cells were exposed to increasing concentrations of **A.** TFP or **B.** TMX, with or without of TRA-8 (0.5 μg/ml), a) Apoptosis was analyzed at 24 hours after treatment (*n* = 3, *p < 0.01; **p < 0.001). b) Western blot analysis of caspase-8, caspase-3 and GAPDH at 8 hours after treatment. Representative blots of three independent experiments are shown.

Using several pancreatic cancer cell lines, we have demonstrated that the expression of DR5 does not consistently correlate with the resistance to TRA-8 [27]. Our previous studies have shown that CaM antagonists promote Fas death receptor-induced apoptosis via regulating the apoptotic/survival signals in the DISC [19]. To determine whether CaM may also play a role in TRA-8-induced apoptosis, we characterized the effects of two CaM antagonists, TFP and TMX, on TRA-8-induced apoptosis in PANC-1 cells (Figure 1). At the concentrations tested, up to 30 μM, neither TFP (Figure 1Aa) nor TMX (Figure 1Ba) induced apoptosis of  PANC-1 cells. In contrast, TFP and TMX enhanced TRA-8-induced apoptosis in concentration-dependent manners (Figure 1Aa & 1Ba). A time-dependent effect of TMX on TRA-induced apoptosis was also demonstrated (Supplementary Figure 1). Consistently, TFP and TMX dramatically enhanced TRA-8-induced activation of caspase-8 and its downstream apoptotic effector caspase-3, as indicated by the respective cleaved forms (Figure 1Ab & 1Bb).

### Caspase-8 inhibition blocks the effects of CaM antagonists on TRA-8-induced apoptosis

**Figure 2:**
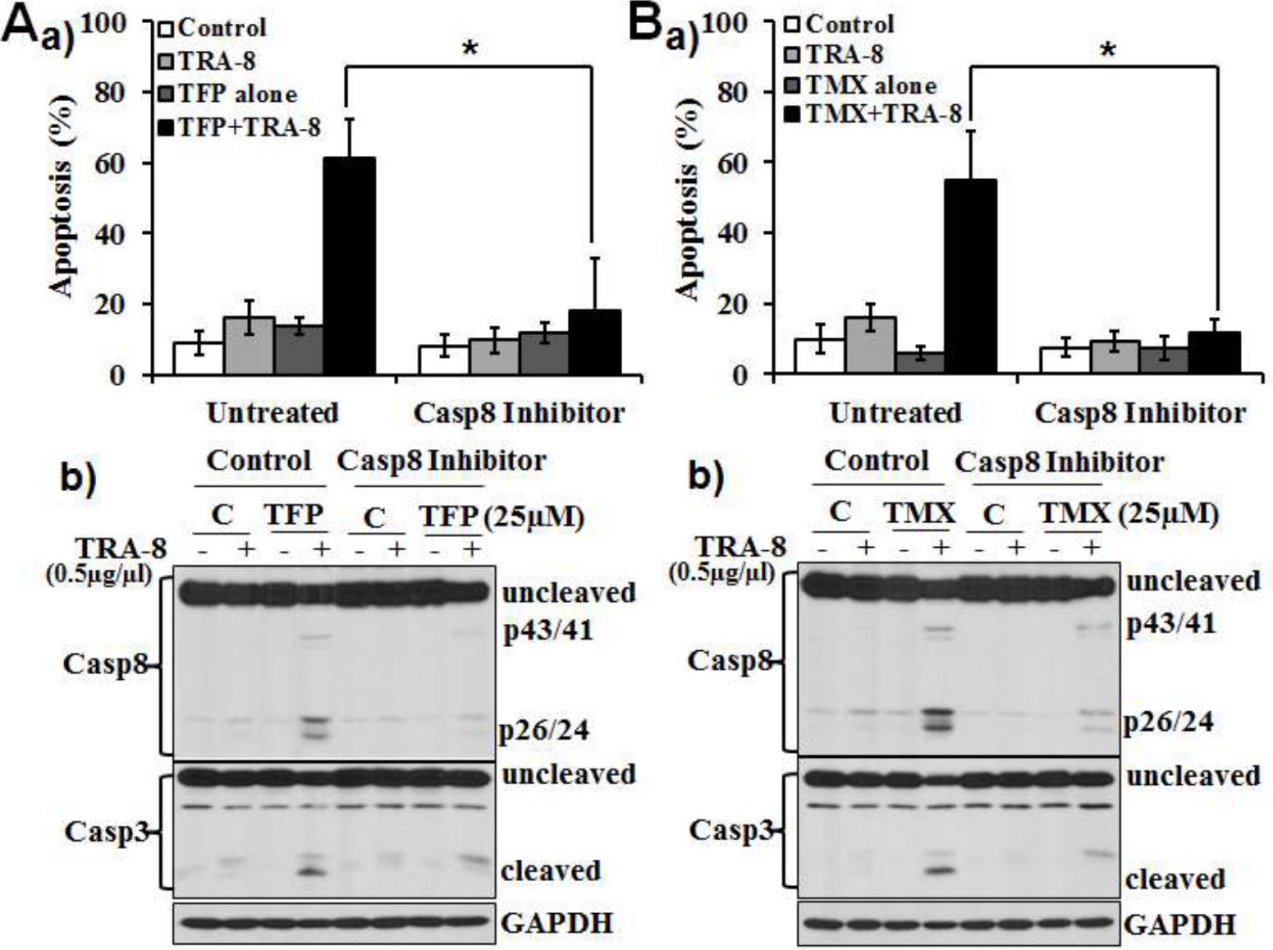
Inhibition of caspase 8 blocks the effect of TFP or TMX on TRA-8-induced apoptosis PANC-1 cells were exposed to **A.** TFP (25 μM) or **B.** TMX (25 μM) alone, TRA-8 (0.5 μg/ml) alone or combined TFP or TMX with TRA-8, with or without pretreatment of caspase-8 inhibitor (Casp8 Inhibitor, Z-IETD-FMK, 20 μmol/L). a) Apoptosis was analyzed at 24 hours after treatment (*n* = 3, *p < 0.001). b) Western blot analysis of caspase-8, caspase-3 and GAPDH at 8 hours after treatment. Representative blots of three independent experiments are shown.

As caspase-8 activation is a key initial molecular event that leads to death receptor-activated apoptosis, we determined whether inhibition of caspase-8 may block the effects of CaM antagonists on TRA-8-induced apoptosis. Z-IETD-FMK, a caspase-8 inhibitor, markedly attenuated apoptosis induced by TRA-8 combined with TFP or TMX (Figure 2Aa & 2Ba). Western blot analysis further determined that TFP and TMX-enhanced activation of caspase-8 (Figure 2Ab & 2Bb, Control) were inhibited by Z-IETZ-FMK (Figure 2Ab & 2Bb, Casp8 Inhibitor). Decreased activation of caspsae-8 was associated with inhibition of caspase-3 activation. Altogether, these results demonstrate that CaM antagonists-enhanced TRA-8-apoptosis of the resistant PANC-1 pancreatic cells is mediated, at least in part, by the activation of caspase-8.

### CaM antagonists increase activation of caspase-8 and decrease CaM and Src in the DISC

**Figure 3:**
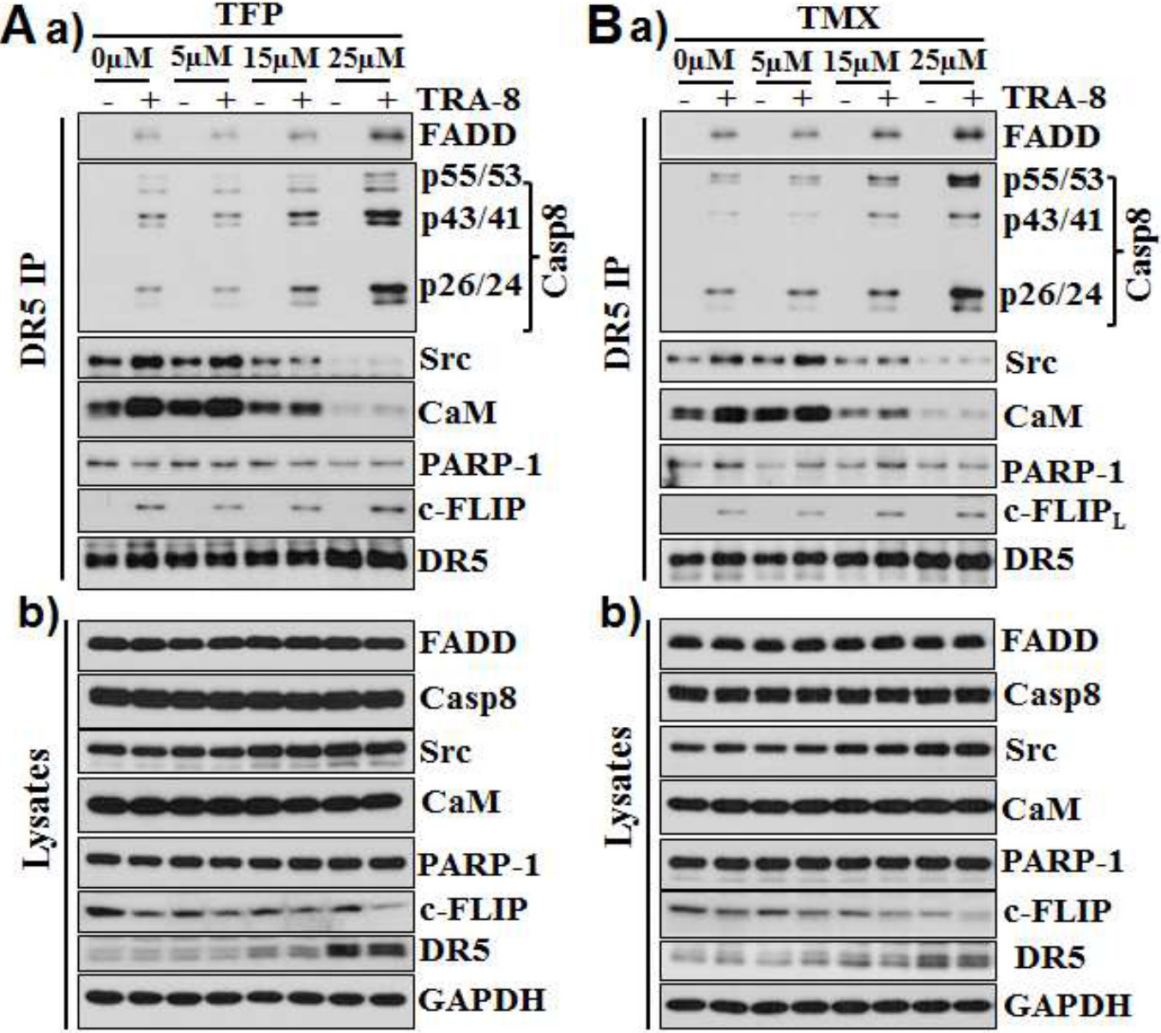
CaM antagonists increase activation of caspase-8 and decrease CaM and Src in the DISC PANC-1 cells were exposed **A.** TMX or **B.** TFP at the indicated concentrations for 16 hours; cells were then treated with TRA-8 (1 μg/ml) for 1 hour. a) Immunoprecipitation of DR5-associated DISC was performed using anti-DR5 antibody. Western blot analysis of the recruitment of FADD, caspase-8, Src, CaM, PARP-1 and FLIP in the DISC. b) Western blot analysis of the expression of FADD, caspase-8, Src, CaM, PARP-1, FLIP and DR5 in cell lysates. The expression of GAPDH was used a loading control. Representative blots from at least three independent experiments are shown.

We have previously shown that recruitment of the poly-ADP-riboso polymerase (PARP-1) into the TRA-8-activated DISC inhibits caspase-8 activation in the DISC, which contributes to the resistance of PANC-1 to TRA-8-induced apoptosis [27]. To determine whether the effects of CaM antagonists on caspase-8 activation were mediated by its regulation of PARP-1, we analyzed the expression and recruitment of PARP-1 in the TRA-8 activated DISC. Neither TFP nor TMX affected PARP-1 expression (Figure 3Ab & 3Bb, cell lysates) or the recruitment of PARP-1 into the DISC (Figure 3Aa & 3Ba, DR5 IP). Therefore, increased activation of caspase-8 in the DISC by TMX and TFP was not due to their effects on PARP-1. Further analysis of the DR5-associated DISC identified the interaction of DR5 with CaM under basal conditions, which was increased upon TRA-8 stimulation (Figure 3A & 3B). The CaM/DR5 interaction was markedly inhibited by the CaM-antagonists, TFP and TMX (Figure 3Aa & 3Ba, DR5 IP). In addition, TFP and TMX inhibited the DISC recruitment of Src, a CaM-associated survival signal in pancreatic cancer cells that we have previously reported [19]. Of note, the expression of Src was not affected by TFP or TMX. The recruitment of another survival signal, FLIP, into the DISC was not affected by TFP or TMX, despite of some decrease in FLIP protein in cells treated with high doses of TFP or TMX. Notably, increased expression of DR5 was evident in cells exposed to 25 μM of TFP or TMX (Figure 3Ab & 3Bb, cell lysates).

### CaM antagonists induce the expression of DR5

**Figure 4:**
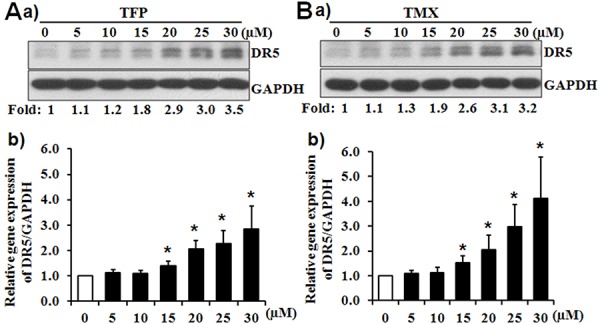
CaM antagonists induce the expression of DR5 PANC-1 cells were exposed to TFP or TMX at indicated concentrations of **A.** TFP or **B.** TMX for 16 hours. The expression of DR5 was determined by a) Western blot analysis; and b) Real-time PCR. a) The expression of GAPDH was used as a loading control. Representative blots from three independent experiments are shown. Numbers below depict the fold induction of DR5 by TFP or TMX at the indicated concentrations compared with that in the control cells (0 μM), which is defined as 1. b) Results shown are fold induction of DR5 mRNA expression, normalized by the expression of GAPDH, induced by TFP or TMX at indicated concentrations compared with that in the control cells, which is defined as 1 (*n* = 3, *p < 0.05).

To further characterize the effects of CaM antagonists on the expression of DR5, we determined the expression of DR5 in PANC-1 cells in response to serial concentrations of TFP or TMX (Figure 4). Western blot analysis demonstrated that either TFP or TMX dose-dependently increased the expression of DR5 protein (Figure 4Aa, 4Ba). In addition, TFP and TMX induced the expression of DR5 mRNA in a dose-dependent manner (Figure 4Ab, 4Bb). The expression of the other TRAIL death receptor, DR4, was not affected by TFP or TMX (data not shown). Furthermore, TMX was also found to induce the expression DR5 in Suit-2 cells (Supplementary Figure 2), another TRA-8 resistant pancreatic cancer cells that we have previously studied [27].

### Identification of CaM antagonists-responsive domain on DR5 gene

**Figure 5:**
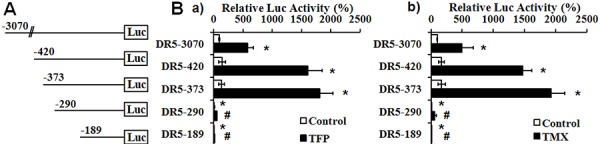
Identification of CaM antagonists-responsive region in the DR5 gene **A.** Schematic map of luciferase reporters containing a serial deletion of the 5′-flanking regions of the DR5 gene. **B.** Relative luciferase activities of the reporters in response to a) TFP and b) TMX. PANC-1 cells that were co-transfected with the indicated luciferase reporters and a control renilla luciferase reporter were exposed to 25 μM of a) TFP or b) TMX for 16 hours. The luciferase activity of DR5-3070 at basal conditions is defined as 100%. TFP or TMX-induced luciferase activities of each reporter, normalized by the renilla luciferase activity, are shown (*n* = 3, *p < 0.001 compared with DR5-3070 at basal condition; and #*p* < 0.001, compared with DR5-373 treated with TFP or TMX).

To determine the mechanisms underlying CaM antagonist-induced DR5 mRNA expression, we characterized the effects of CaM antagonists on the transcriptional activity of the DR5 gene, using luciferase reporters containing a series of deletion mutants of the 5′-flanking regions of DR5 (Figure 5A). TFP and TMX markedly induced luciferase activity in PANC-1 cells transfected with luciferase reporter constructs DR5-3070, 420 and 373 (Figure 5Ba, 5Bb). However, TFP and TMX-induced luciferase activity was dramatically decreased in the DR5-290 and 189 transfected cells, and the basal luciferase activity in these cells was also significantly reduced compared with that in the DR5-3070 transfected cells (Figure 5B & 5C). Taken together, the results indicate that the region located between −290 to −373 on the DR5 gene is responsible for CaM antagonist-induced DR5 mRNA expression.

**Figure 6:**
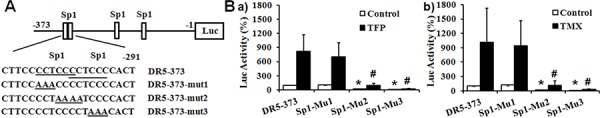
CaM antagonists-responsive region in the DR5 gene **A.** Schematic map of the putative Sp1 binding elements in the CaM antagonist-responsive region and mutation strategies. The two putative Sp1 binding elements and the three mutants on the DR5-373 luciferase reporter are underlined. **B.** The effects of the mutations on the putative Sp1 binding elements on CaM antagonists-induced DR5 expression. PANC-1 cells transfected with luciferase reporter DR5-373 or its mutants were treated with 25 μM of a) TFP or b) TMX for 16 hours. Results shown are relative luciferase activity, after normalized by the renilla luciferase activity, compared with wild type DR5-373 in control condition, defined as 100% (*n* = 3, *p < 0.001, compared with DR5-373 at control condition; #*p* < 0.001, compared with DR5-373 treated by TFP or TMX).

Analysis of the sequence between −290 to −373 of DR5 gene identified two putative binding elements for the transcription factor specificity protein 1 (Sp1) [28], which are located between −295 to −300bp and −300 to −305bp (Figure 6A, DR5-373 underline). Mutation of 3 nucleotides in the first putative Sp1 binding site (DR5-373-mut1) did not affect the luciferase activity under basal conditions and upon TFP or TMX stimulation (Figure 6B). In contrast, mutations of 3 cytosines overlapping the two putative Sp1-binding elements (DR5-373-mut2) or in the second putative Sp1-binding site (DR5-373-mut2) abolished the induction of luciferase activity by TFP (Figure 6Ba) or TMX (Figure 6Bb). Accordingly, the second putative Sp1 binding region locating between −295 to −300bp on the DR5 gene is responsive for CaM antagonist-induced DR5 gene expression.

### TMX induces DR5 expression and enhances efficacy of TRA-8 therapy on pancreatic cancer tumorigenesis

**Figure 7:**
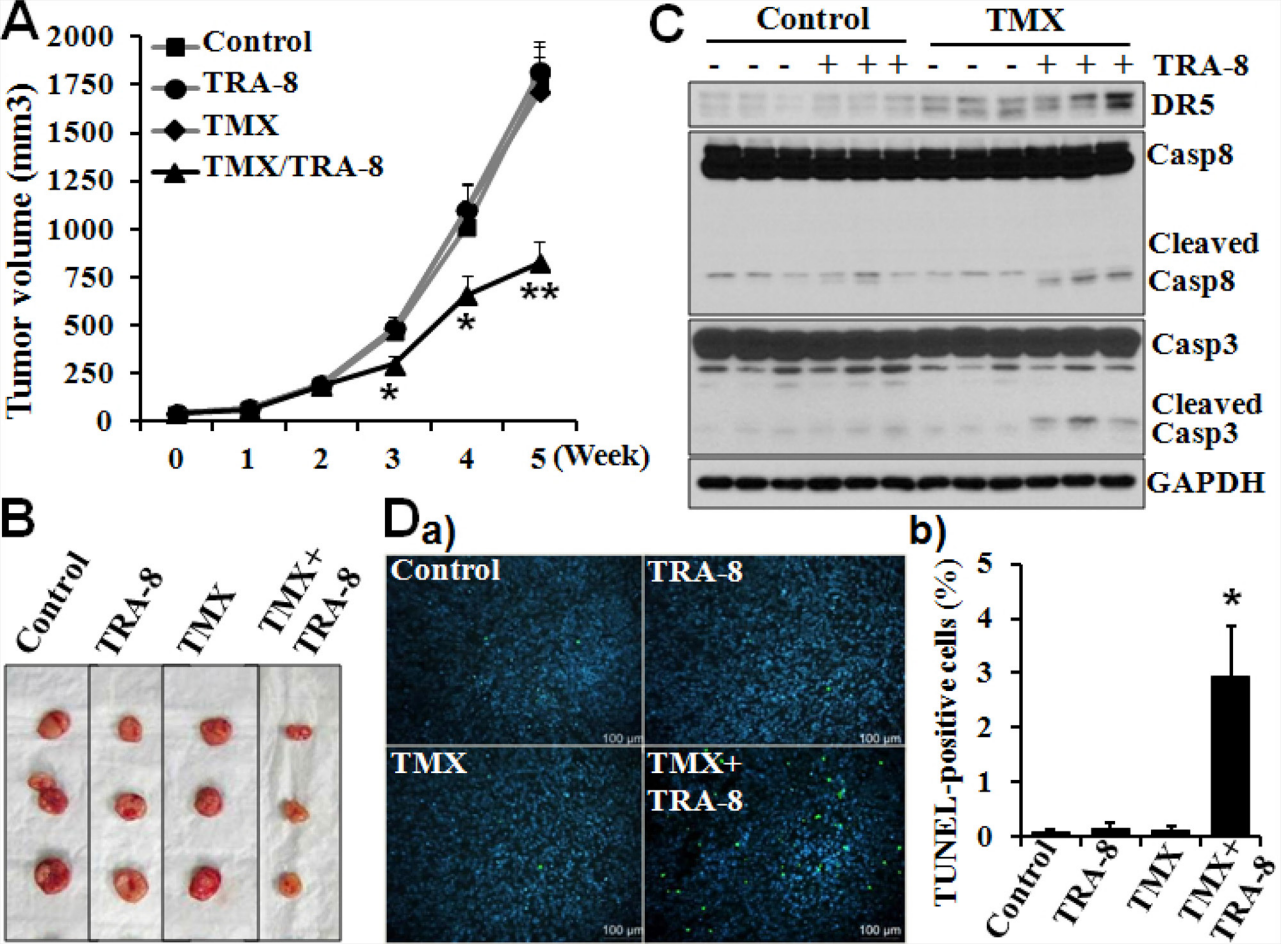
Tamoxifen enhances the efficacy of TRA-8 on pancreatic cancer cell tumorigenesis in mice Tumorigensis of PANC-1 cells in nude mouse xenograft model as described in “materials and methods”. **A.** Tumor volumes in each group for 5 weeks after treatment are shown. Results are presented as mean ± SE, *p < 0.05 and **p < 0.01 compared with TRA-8 alone, *n* = 16 in each group). **B.** Representative tumors from each group at 5 weeks after treatment. **C.** Western blot analysis of the expression of DR5 and activation of caspase-8 and caspase-3 in isolated tumors. Results shown in B and C are from 3 representative tumors in each group. **D.** Cell death, analyzed by TUNEL staining, in tumors a) and b) Quantitative analysis of TUNEL-positive cells as percentage of total cells in the tumor sections (*n* = 5 tumors in each group, *p < 0.001).

To further determine whether CaM antagonists affect DR5 expression and TRA-8-induced apoptosis *in vivo*, we characterized the therapeutic efficacy of TRA-8, TMX and TRA-8 plus TMX on PANC-1 tumorigenesis a mouse xenograft model. Similar to our previous observation [27], TRA-8 alone was not effective in inhibiting PANC-1 tumorigenesis (Figure 7A, 7B). TMX by itself did not affect tumor growth (Figure 7A, 7B), which is consistent with the *in vitro* observation that TMX alone did not induce apoptosis (Figure 1B). In contrast, TMX significantly enhanced the efficacy of TRA-8 treatment (Figure 7A, 7B). The inhibitory effects of TRA-8 combined with TMX on tumor growth was pronounced at 3 weeks and further enhanced at 5 weeks (Figure 7A). The effects of TMX to increased DR5 expression of tumors was determined by Western blots analysis of tumor tissues (Figure 7C). Increased activation of caspase-8 and caspase-3, as indicated by cleaved caspase-8 and 3, was determined in tumors treated with TRA-8 combined with TMX (Figure 7C), indicating increased tumor cell apoptosis. Furthermore, TUNEL staining of tumor sections demonstrated significant increases in cell death in the tumors from mice treated with the combination of TMX and TRA-8 (Figure 7D). These results demonstrate that TMX also increases DR5 expression and induces apoptosis in tumors, and thus enhancing the efficacy of TRA-8 therapy.

## DISCUSSION

Activating TRAIL-induced apoptosis for cancer therapy has been actively investigated in a variety of cancers (http://www.clinicaltrials.gov). However, phase I–II clinical trials with recombinant TRAIL and agonistic antibodies for the TRAIL receptors, DR4 and DR5, have shown only isolated responses and limited overall effects on tumor progression [11, 12, 29, 30], indicating resistance of tumor cells to TRAIL-induced cell death. The present work demonstrate that CaM antagonists enhance TRAIL-induced apoptosis in resistant pancreatic cancer cells via novel mechanisms, which supports the use of these readily available drugs as promising interventions to improve the efficacy of TRAIL therapy.

Using two potent CaM antagonists, TFP and TMX, we have characterized the ability of CaM antagonists to enhance TRA-8-induced apoptosis in two TRA-8-resistant pancreatic cancer cell lines. The effects of CaM antagonists on TRA-8-induced apoptosis is not due to their toxicity or via stimulation of intrinsic apoptotic signaling pathways as seen in other cancer cells [31], as neither TFP nor TMX alone was found to affect the survival or apoptosis of the TRA-8 resistant pancreatic cancer cell lines. In contrast, CaM antagonists enhanced TRA-8-induced apoptosis by modulating the extrinsic apoptosis pathways via increasing the DR5-associated DISC recruitment and activation of caspase-8. Such an observation is similar to our previous report that CaM antagonists promote Fas death receptor-induced apoptosis via the extrinsic apoptosis pathways mediated by caspase-8 activation [22, 32]. Furthermore, TMX markedly enhanced the efficacy of TRA-8 therapy on tumorigenesis of the resistant PANC-1 pancreatic cancer cells *in vivo*, which was associated with increased expression of DR-5 and increased activation of the caspase-8.

Analysis of the DR5-associated DISC identified the interaction of CaM and DR5 in the pancreatic cancer cell lines that was increased in response to TRA-8. These results in pancreatic cancer cells differ from our previous report in non-cancerous jurkat cells in which CaM did not interact with TRAIL death receptors [33]. In the Fas-activated DISC, we have demonstrated that the recruitment of CaM into the DISC facilitates the recruitment of survival signals, including the FLIP and Src [19–22, 32], which may contribute to the effects of CaM antagonists on enhancing Fas-induced apoptosis [21, 22]. Similarly, FLIP and Src were found to be recruited into the DR5-associated DISC. However, CaM antagonists inhibit the recruitment of both FLIP and Src in the Fas-activated DISC, whereas CaM antagonists only inhibited the recruitment of Src, but not FLIP, into the DR5-associated DISC. Considering also that the expression of FLIP is very low in TRA-8-resistant PANC-1 cells [27], these results suggested that FLIP may not play a key role in mediating the effects of CaM antagonists on enhancing TRA-8-induced apoptosis.

The precise function of Src in the DR5-associated DISC has not been determined yet. We have demonstrated that CaM-mediated Src recruitment into the Fas-activated DISC in pancreatic cancer cells is associated with Src phosphorylation/activation and cell survival [19]. In Hela cells, Src activation has been demonstrated to inhibit caspase-8 activation, via phosphorylating caspase-8 at tyrosine 380 that inhibits caspase-8 cleavage [34]. In addition, Src inhibition is associated with increased caspase-8 cleavage, which contributes to increased apoptosis in TRAIL resistant hepatic carcinoma cells [35]. Accordingly, recruitment of Src, via CaM, into the death receptor-activated DISC may provide the proximity for Src to phosphorylate caspase-8, which inhibits caspase-8 cleavage and activation as seen in Hela cells. Consistently, we have found that CaM antagonist, TFP, decreases CaM binding to Src, which inhibits Fas-induced recruitment of Src into the DISC and Src phosphorylation at tyrosine 416 that are key to its activation [19]. Therefore, inhibition of Src recruitment and activation in the DR5-associated DISC by CaM antagonists may contribute to CaM antagonist enhancement of TRA-8-induced apoptosis. Additionally, we have recently reported that TRA-8-induced DISC recruitment of PARP-1 regulates caspase-8 activation in the DISC [27], which is accompanied by enhanced sensitivity of pancreatic cancer cell to TRA-8-induced apoptosis. However, CaM antagonists did not affect PARP-1 expression or the recruitment of PARP-1 into TRA-8-induced DISC. Thus, CaM antagonists-enhanced caspase-8 activation and apoptosis is not mediated by PARP-1.

Our studies have further identified a novel mechanism whereby CaM antagonists enhance TRA-8-induced apoptosis by inducing the expression of DR5. Although the correlation between the basal expression levels of the functional death receptors, DR4 or DR5, and the sensitivity of cancer cells to TRAIL treatment has not been demonstrated [27, 36, 37], our results are consistent with previous observations that upregulation of DR4 and DR5 enhance TRAIL-induced apoptosis in a variety of cancers [38–40]. Furthermore, results from our studies have provided new mechanistic insights into CaM antagonist-indued DR5 expression. We demonstrated that CaM antagonists enhanced the expression of DR5 in pancreatic cancer cells, but not the other TRAIL functional receptor, DR4. The CaM antagonist-responsive region was localized between −295 and −300 bp in the 5′ flanking region of the DR5 gene, a putative binding element of the specificity protein 1 (Sp1). In addition to Sp1 [41–43], other transcription factors that have been reported to modulate DR5 transcription include the nuclear factor κB [44], CCAAT/enhancer-binding protein homologous protein [45], activator protein 1 [46] and Yin Yang 1 [47]. However, no putative binding elements for the other transcription factors were identified in the CaM antagonist-responsive region. Of note, previous studies have demonstrated that activation of Sp1 is unique for transcriptional regulation of DR5 without affecting DR4 expression in other cancer cells, including colon [41], hepatoma [43] and ovarian cancer cells [42]. Consistently, site-direct mutagenesis studies further support a unique and important role of Sp1 in mediating CaM-antagonist-induced upregulation of DR5.

In summary, we have demonstrated that CaM antagonists, TFP and TMX, enhance TRA-8-induced apoptosis in TRA-8-resistant pancreatic cancer cell lines. CaM antagonists induce DR5 expression via two putative Sp1 binding elements spanning −295 to −305 bp of DR5 gene, and increase recruitment and activation of apoptotic signal, caspase-8, and decrease survival signals, CaM and Src, in the TRA-8-activated DISC. This novel regulatory role of CaM in the DR5-associated DISC may present a unique opportunity for the use of these readily available and well tolerated CaM antagonists, TMX and TFP, in combination with DR5 agonists to enhance the therapeutic efficacy of TRAIL-resistant pancreatic cancer.

## MATERIALS AND METHODS

### Cell culture, antibodies, and reagents

The human pancreatic cancer cell lines PANC-1 and Suit-2 were purchased from the American Type Culture Collection (ATCC, Manassas, VA). PANC-1 cells were grown in Dulbeccos' Modified Eagle's Media (DMEM; Invitrogen, Carlsbad, CA)) and Suit 2 cells were grown in RPMI 1640 supplemented with penicillin (5 units/mL), streptomycin (5 μg/mL), and 10% heat-inactivated FBS.

DR5 agonist antibody, TRA-8, was generated as previously described [48]. All antibodies used were commercially available, including anti-caspase-8 (BD Bioscience, San Jose, CA), anti-caspase-3 (Enzo Life, Plymouth Meeting, PA), anti-FADD and anti-CaM (Millipore, Billerica, MA), Src (Cell Signaling Technology, Danvers, MA), anti-DR5 (Prosci, Poway, CA), anti-c-FLIP (Enzo Life, Farmingdale, NY) and anti-GAPDH (Santa Cruz Biotech, Santa Cruz, CA).

Tamoxifen (TMX) and Trifluoperazine (TFP) were obtained from Sigma-Aldrich (St. Louis, MO). Protein G-agarose and Lipofectamine 2000 were from Invitrogen (Carlsbad, CA). Caspase-8 Inhibitor, Z-IETD-FMK, was from R&D system. The dual-luciferase activity reporter system was purchased from Promega.

### Assessment of apoptosis

Cells were exposed to TRA-8, TMX and TFP for the times and concentrations indicated in the figure legends. Apoptosis was determined by Annexin V-fluorescein isothiocyanate (FITC) and propidium iodide (PI) staining (BD Bioscience, San Jose, CA) and analyzed by flow cytometry (BD Biosciences).

### Western blot analysis

Proteins were extracted, quantified with a BCA protein assay kit (Thermo Scientific, Waltham, MA), separated by SDS-PAGE and transferred to Immobilon P membranes (Millipore, Billerica, MA) as described previously [27]. Membranes were blocked in 5% nonfat milk and incubated with primary antibodies overnight at 4°C. Horseradish peroxidase-conjugated secondary antibodies in the blocking buffer were incubated for 1 h at room temperature. Signals were detected using Immobilon Western chemiluminescent horseradish peroxidase substrate detection kit (Millipore, Billerica, MA).

### Immunoprecipitation

Immunoprecipitation of DISC proteins was performed as we described previously [27]. Cells were suspended and incubated with TRA-8 for 1 hour at 37°C. Protein extracts (1000 μg) was then incubated with 1 μg of anti-DR5 antibody for 1 hour and subsequently incubated with 50 μL of 1:1 slurry of protein G-agarose beads overnight at 4°C. Beads were washed, and 20 μL 2 × Laemmli sample buffer was added to the beads followed by heating at 95°C for 5 minutes and chilling on ice. After brief centrifugation, proteins in the supernatant were analyzed by Western blotting with indicated antibodies.

### Real-time PCR analysis

Total RNA was extracted with Trizol reagents according to the manufacturer's instructions (Invitrogen Carlsbad, CA). The reverse transcription reaction was performed on 1 μg of total RNA using the First Strand cDNA Synthesis Kit (Fermentas, Glen Burnie, MD). Real-time PCR for DR4, DR5 and GAPDH was performed and analyzed using SsoFast EvaGreen Supermix (Bio-Rad, Hercules, CA) in a Bio-Rad CFX96-Cycler as we previously described [49].

### Luciferase reporter assay

DR5 promoter activity was determined using a dual-luciferase activity reporter system. The promoter report plasmids containing a 5′-flanking region of DR5 gene from −1~-373 (pGL3-DR5-373), −1~-420(pGL3-DR5-420) and −1~-3070 (pGL3-DR5-3070) were kindly provided by Dr. Sun SY [49]. A serial deletion and point mutations were generated by PCR with specific primers using the plasmid pGL3-DR5-373 as a template. The amplified fragments were inserted into the KpnI and BglII restriction sites of the pGL3-basic reporter vector. Specific primers include the reverse primer, 5′-CTTAAGATCTGGCGGTAGGGAACGCTCTTATAG TC-3′; and the forward primers: 5′-CTTAGG TACCTGGACGCGCTTGCGGAGGATTGCGT-3′ (pG L3-DR5–290); 5′-CTTAGGTACCCGAATGAC GCCTGC CCGGAGGCAGT-3′ (pGL3-DR5-189); and 5′-CTTAG GTACCAAGTCAGCCTGGACACA TAAATCAG-3′ (pG L3-DR5-142). Point mutations were made by replacing the nucleic acids with adenine using the QuikChange II Site-Directed Mutagenesis kit (Agilent Technologies, Santa Clara, CA) and confirmed by sequence analysis. Specific primers are 5′-5′-TTAGTTCCGGTCCCTTCCAAACCCC TCCCCACTTGGACG-3′ and 5′-CGTCCAAGTGGGGA GGGGTTTGGAAGGGACCGGAACTAA-3′ (DR5-Sp1 -Mutant 1, −303 to −305); 5′-GTTCCGGTCCCTTCCC CTAAAATCCCCACTTGGACGCGC T-3′ and 5′-AGCG CGTCC AAGTGGGGATTTT AGGGGAAGGGACCG GAAC-3′ (DR5-Sp1-Mutant 2, −299 to −302); 5′-GG TCCCTTCCCCTCCCCTAAACACTTGGACGCGCTT GCGGA-3′ and 5′-TCCGCAAGCGCGTCC AAGTGT TTA GGGGAGGGGAAGGGACC-3′(DR5-Sp1-Mutant3, - 295 to −297).

To determine luciferase activity, PANC-1 Cells at 70–80% confluence were co-transfected with pGL3-DR5 reporter plasmids and a plasmid expressing the venilla luciferase (as a control for transfection efficiency) using Lipofectamine 2000. Media were changed 24 hours after transfection, and cells were treated with TMX and TFP for 16 hours. Luciferase activity was measured with Dual-Luciferase Reporter Assay System [50].

### Mouse xenograft model

The animal protocol was approved by the Institutional Animal Care and Use Committee at the University of Alabama at Birmingham. Male athymic *nu*/*nu* mice (6 weeks old, NCI-Frederick) were used for tumor inoculation as we previously reported [27]. Briefly, PANC-1 cells (2 × 10^6^ in 200 mL PBS/site) were inoculated subcutaneously into the both flanks of mice. Five days after tumor inoculation, mice were divided into four groups(8 mice/group): a control group injected with 0.9% sodium chloride and three treatment groups intraperitoneally injected with TMX (15 mg/kg, 2 consecutive days/week), TRA-8 (200 μg/mice, once/week), or the combination (TMX plus TRA-8). The tumor size was measured every week and tumor volumes were determined using the formula volume = length × width^2^/2. At the end of the experiment, tumors were removed from mice and homogenized for Western blot analysis.

### TUNEL staining

TUNEL staining was performed on tumor sections (7 μm) (DeadEnd Fluorometric TUNEL System; Promega) to determine cell death, and DAPI staining (4′, 6-diamidine-2-phenylindole dihydrochloride) was used to identify nuclei. Stained specimens were examined microscopically (Leica M165 FC). For quantitative analysis, cell numbers were counted under a microscope (× 200). Four fields in each slide were counted and the percentage of apoptotic cells was determined.

### Statistical analysis

Results are generally expressed as means ± SD unless specified. Differences between 2 groups were identified with the Student *t* test. For multiple groups, one-way ANOVA and Student–Newman–Keuls tests were conducted to identify differences. Significance was defined as *P* < 0.05.

## SUPPLEMENTARY FIGURES


