# Supplementary material for: Calmodulin antagonists promote TRA-8 therapy of resistant pancreatic cancer
Source: Oncotarget. 2015 Jul 11;6(28):25308–19. doi: 10.18632/oncotarget.4490 (PMC4694833; doi:10.18632/oncotarget.4490)
Supplement: Supplementary file 1 [file oncotarget-06-25308-s001.pdf]

SUPPLEMENTARY FIGURES

TMX enhances TRA-8-induced apoptosis in a time-dependent manner. The effect of TMX on TRA-8-induced apoptosis was determined at different time points. Significant increase in TRA-8-induced apoptosis by TMX was demonstrated at 8 hours, which was further enhanced at 16 and 24 hours.

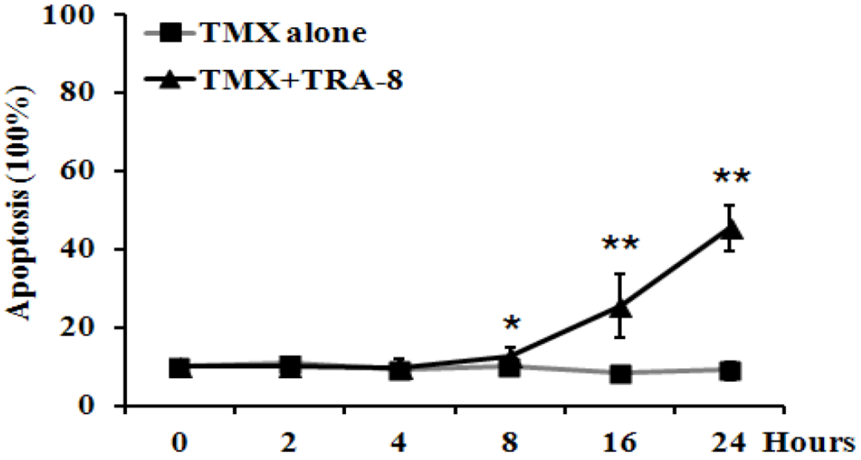

Supplementary Figure 1: Time-dependent effects of TMX on TRA-8-induced apoptosis. PANC-1 cells were exposed to TRA-8 with or without TMX (25 µM), apoptosis was determined at indicated time points. Results from 3 independent experiments performed in triplicates are shown (*$p < 0.05$ and **$p < 0.001$ compared with 0 hour).

TMX induces the expression of DR5 in Suit-2 pancreatic cancer cells. The effects of CaM antagonists on the expression of DR5 were further determined in Suit-2 cells, another TRA-8 resistant pancreatic cancer cells, which we have previously studied [1]. Similar to our observation in the PANC-1 pancreatic cancer cells, we found that TMX dose-dependently induced the expression of DR5 in Suit-2 cells.

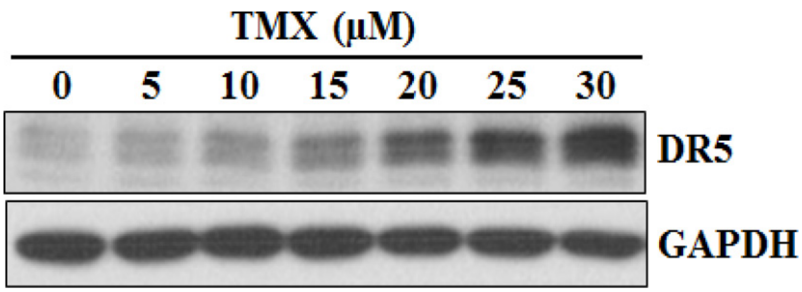

Supplementary Figure 2: TMX induced DR5 expression in Suit-2 pancreatic cancer cells. Suit-2 cells were exposed to TMX at indicated concentrations for 16 hours; Western blotting analysis was performed using a specific antibody for DR5 expression. The expression of GAPDH was used as a loading control.

REFERENCE

1. Yuan K, Sun Y, Zhou T, McDonald J, Chen Y. PARP-1 regulates resistance of pancreatic cancer to TRAIL therapy. Clinic cancer research. 2013; 19:4750–9.
